# Investigating the prevalence of *MFN2* mutations in amyotrophic lateral sclerosis: insights from an Italian cohort

**DOI:** 10.1093/braincomms/fcae312

**Published:** 2024-09-23

**Authors:** Elena Abati, Delia Gagliardi, Arianna Manini, Roberto Del Bo, Dario Ronchi, Megi Meneri, Francesca Beretta, Annalisa Sarno, Federica Rizzo, Edoardo Monfrini, Alessio Di Fonzo, Maria Teresa Pellecchia, Alberto Brusati, Vincenzo Silani, Giacomo Pietro Comi, Antonia Ratti, Federico Verde, Nicola Ticozzi, Stefania Corti

**Affiliations:** Dino Ferrari Centre, Department of Pathophysiology and Transplantation (DEPT), Università degli Studi di Milano, 20122 Milan, Italy; Neurology Unit, Fondazione IRCCS Ca’ Granda Ospedale Maggiore Policlinico, 20122 Milan, Italy; Dino Ferrari Centre, Department of Pathophysiology and Transplantation (DEPT), Università degli Studi di Milano, 20122 Milan, Italy; Dino Ferrari Centre, Department of Pathophysiology and Transplantation (DEPT), Università degli Studi di Milano, 20122 Milan, Italy; Dino Ferrari Centre, Department of Pathophysiology and Transplantation (DEPT), Università degli Studi di Milano, 20122 Milan, Italy; Dino Ferrari Centre, Department of Pathophysiology and Transplantation (DEPT), Università degli Studi di Milano, 20122 Milan, Italy; Neurology Unit, Fondazione IRCCS Ca’ Granda Ospedale Maggiore Policlinico, 20122 Milan, Italy; Department of Neurosciences, Psychology, Drug Research and Child Health (NEUROFARBA), Università degli Studi di Firenze, 50139 Firenze, Italy; Dino Ferrari Centre, Department of Pathophysiology and Transplantation (DEPT), Università degli Studi di Milano, 20122 Milan, Italy; Neurology Unit, Fondazione IRCCS Ca’ Granda Ospedale Maggiore Policlinico, 20122 Milan, Italy; Dino Ferrari Centre, Department of Pathophysiology and Transplantation (DEPT), Università degli Studi di Milano, 20122 Milan, Italy; Neurology Unit, Fondazione IRCCS Ca’ Granda Ospedale Maggiore Policlinico, 20122 Milan, Italy; Neurology Unit, Fondazione IRCCS Ca’ Granda Ospedale Maggiore Policlinico, 20122 Milan, Italy; Department of Medicine, Surgery and Dentistry ‘Scuola Medica Salernitana’, Neuroscience Section, Università degli Studi di Salerno, 84081Salerno, Italy; Department of Brain and Behavioral Sciences, Università degli Studi di Pavia, 27100 Pavia, Italy; Dino Ferrari Centre, Department of Pathophysiology and Transplantation (DEPT), Università degli Studi di Milano, 20122 Milan, Italy; Department of Neurology and Laboratory of Neuroscience, IRCCS Istituto Auxologico Italiano, 20149 Milan, Italy; Dino Ferrari Centre, Department of Pathophysiology and Transplantation (DEPT), Università degli Studi di Milano, 20122 Milan, Italy; Neurology Unit, Fondazione IRCCS Ca’ Granda Ospedale Maggiore Policlinico, 20122 Milan, Italy; Department of Neurology and Laboratory of Neuroscience, IRCCS Istituto Auxologico Italiano, 20149 Milan, Italy; Department of Medical Biotechnology and Translational Medicine, Università degli Studi di Milano, 20133 Milan, Italy; Dino Ferrari Centre, Department of Pathophysiology and Transplantation (DEPT), Università degli Studi di Milano, 20122 Milan, Italy; Department of Neurology and Laboratory of Neuroscience, IRCCS Istituto Auxologico Italiano, 20149 Milan, Italy; Dino Ferrari Centre, Department of Pathophysiology and Transplantation (DEPT), Università degli Studi di Milano, 20122 Milan, Italy; Department of Neurology and Laboratory of Neuroscience, IRCCS Istituto Auxologico Italiano, 20149 Milan, Italy; Dino Ferrari Centre, Department of Pathophysiology and Transplantation (DEPT), Università degli Studi di Milano, 20122 Milan, Italy; Neuromuscular and Rare Diseases Unit, Department of Neuroscience, Fondazione IRCCS Ca’ Granda Ospedale Maggiore Policlinico, Milan 20122 Milan, Italy

**Keywords:** mitofusin 2, amyotrophic lateral sclerosis, genetics, mitochondria

## Abstract

The *MFN2* gene encodes mitofusin 2, a key protein for mitochondrial fusion, transport, maintenance and cell communication. *MFN2* mutations are primarily linked to Charcot–Marie–Tooth disease type 2A. However, a few cases of amyotrophic lateral sclerosis and amyotrophic lateral sclerosis/frontotemporal dementia phenotypes with concomitant *MFN2* mutations have been previously reported. This study examines the clinical and genetic characteristics of an Italian cohort of amyotrophic lateral sclerosis patients with rare, non-synonymous *MFN2* mutations. A group of patients (*n* = 385) diagnosed with amyotrophic lateral sclerosis at our Neurology Units between 2008 and 2023 underwent comprehensive molecular testing, including *MFN2*. After excluding pathogenic mutations in the main amyotrophic lateral sclerosis–related genes (i.e. *C9orf72*, *SOD1*, *FUS* and *TARDBP*), *MFN2* variants were classified based on the American College of Medical Genetics and Genomics guidelines, and demographic and clinical data of *MFN2*-mutated patients were retrieved. We identified 12 rare, heterozygous, non-synonymous *MFN2* variants in 19 individuals (4.9%). Eight of these variants, carried by nine patients (2.3%), were either pathogenic, likely pathogenic or variants of unknown significance according to the American College of Medical Genetics and Genomics guidelines. Among these patients, four exhibited a familial pattern of inheritance. The observed phenotypes included classic and bulbar amyotrophic lateral sclerosis, amyotrophic lateral sclerosis/frontotemporal dementia, flail arm, flail leg and progressive muscular atrophy. Median survival after disease onset was extremely variable, ranging from less than 1 to 13 years. This study investigates the prevalence of rare, non-synonymous *MFN2* variants within an Italian cohort of amyotrophic lateral sclerosis patients, who have been extensively investigated, enhancing our knowledge of the underlying phenotypic spectrum. Further research is needed to understand whether *MFN2* mutations contribute to motor neuron disease and to what extent. Improving our knowledge regarding the genetic basis of amyotrophic lateral sclerosis is crucial both in a diagnostic and therapeutic perspective.

## Teaser

Abati *et al*. reported nine patients diagnosed with amyotrophic lateral sclerosis and carrying eight rare variants within the *MFN2* gene, identified among a cohort of 385 amyotrophic lateral sclerosis patients who underwent *MFN2* screening. This study enhances the knowledge regarding the phenotypic spectrum underlying *MFN2* alterations.

## Graphical Abstract

**Graphical Abstract:**
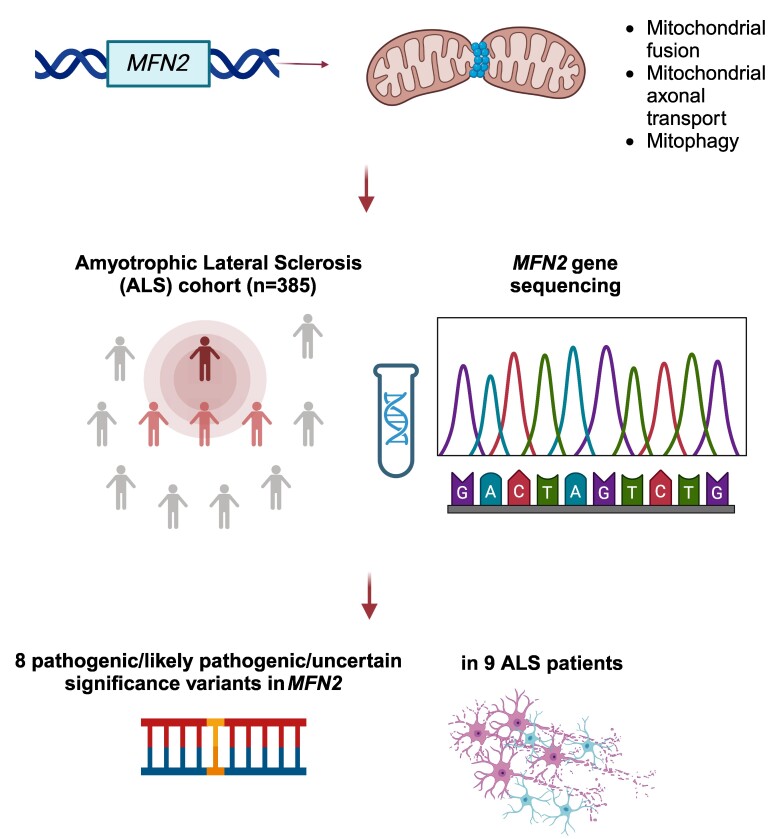

## Introduction

Amyotrophic lateral sclerosis is a devastating neurodegenerative disease characterized by the progressive loss of motor neurons (MNs) in the motor cortex and spinal cord.^1,2^ Sporadic amyotrophic lateral sclerosis, occurring without any family history, represents 85–90% of all cases, with median onset age ranging 58–63 years.^1^ Familial amyotrophic lateral sclerosis accounts for the remaining 10–15% cases, with a slightly younger age at onset (47–53 years).^1,3^ Causative mutations have been identified in more than 50 genes. However, most mutations are found in only four of them (i.e. *C9orf72*, *TARDBP*, *FUS* and *SOD1*), which account for almost 75% familial amyotrophic lateral sclerosis and 20% sporadic amyotrophic lateral sclerosis cases.^1,4,5^ Despite being the most common form of MN disease (MND) in adults, effective treatments for amyotrophic lateral sclerosis, particularly for sporadic forms, remain elusive. In this scenario, the molecular therapy approach is emerging as a compelling and viable strategy, bolstered by the recent approval of tofersen,^6^ which underscores the importance of identifying genetic causes in amyotrophic lateral sclerosis patients.

Lately, a growing number of genes have been linked to both Charcot–Marie–Tooth disease type 2 and amyotrophic lateral sclerosis (i.e. *CHCHD10*, *GARS*, *FIG4*, *KIF5A*, *NEFH*, *VCP* and *SPG11*),^7^ suggesting a genetic overlap between the two diseases, although further studies are needed to confirm this association. Interestingly, a few cases of occurrence of amyotrophic lateral sclerosis/frontotemporal dementia with *MFN2* mutations have been reported in the literature.^8,9^ More recently, Russel *et al*.^10^ reported six rare *MFN2* variants, which were classified as deleterious according to the VAAST (Variant Annotation, Analysis and Search Tool) and Phevor (Phenotype Driven Variant Ontological Re-ranking) software, within a cohort of 140 amyotrophic lateral sclerosis patients, and other 15 variants in the publicly available database ALSdb, composed of ∼2800 amyotrophic lateral sclerosis patients. Autosomal dominant and, to a lesser extent, recessive mutations in *MFN2* are associated with Charcot–Marie–Tooth disease type 2A,^11^ the most frequent axonal subtype of Charcot–Marie–Tooth disease. Charcot–Marie–Tooth disease type 2A has been associated with early-onset, severe motor-predominant polyneuropathy presenting with muscle weakness, atrophy and sensory loss with mainly distal involvement.^12,13^ Moreover, Charcot–Marie–Tooth disease type 2A patients may manifest a wide spectrum of additional symptoms, such as optic atrophy, pyramidal tract alterations and cerebral white matter abnormalities.^12-18^ While there is still no cure for Charcot–Marie–Tooth disease type 2A, our group has been actively involved in the development of gene therapy approaches aimed at modulating mutant/wild-type *MFN2* expression in preclinical models, as described in our recent work,^19^ an approach that could be applied to all *MFN2*-related diseases.

MFN2 is a mitochondrial transmembrane GTPase that plays a crucial role in several mitochondrial processes, including fusion, trafficking, turnover and organelle interactions, ensuring proper mitochondrial shape, function and distribution within cells.^20,21^ The mitochondrial fusion process involves the MFN2 heptad repeat domain and GTP hydrolysis, leading to the binding and oligomer formation of MFN2 and MFN1 proteins on distinct mitochondria, facilitating their fusion into a single mitochondrion. The importance of MFN2 is evident from studies led in *Mfn2* knock-out (KO) mice, which do not survive past midgestation, and homozygous mutants that die postnatally.^20,22^ Intriguingly, in a mouse model of amyotrophic lateral sclerosis, the MFN2 protein was found to interact with TDP-43, a protein involved in the pathogenesis and neuropathology of frontotemporal dementia and amyotrophic lateral sclerosis.^23^

Here, we retrospectively reviewed our cohort of 385 amyotrophic lateral sclerosis patients to investigate the prevalence of rare, non-synonymous *MFN2* variants. Our analysis led to the identification of eight different *MFN2* variants, which were classified as either pathogenic, likely pathogenic or variants of unknown significance (VUS) according to the American College of Medical Genetics and Genomics (ACMG) criteria, in heterozygous state in nine individuals. The observed phenotypes ranged across a wide spectrum of MNDs, including classic amyotrophic lateral sclerosis, amyotrophic lateral sclerosis/frontotemporal dementia and restricted presentations.

## Materials and methods

### Data collection

We recruited a cohort of 385 patients with MND (amyotrophic lateral sclerosis, primary lateral sclerosis and progressive muscular atrophy) according to the El Escorial and Awaji-Shima criteria,^24,25^ among those diagnosed (*n* = 1600) in the MND Clinics of the Neurology Units of Fondazione IRCCS Ca′ Granda Ospedale Maggiore Policlinico of Milan and IRCCS Auxologico of Milan between January 2008 and July 2023. Neurological evaluation and medical chart review were performed by neurologists experienced in neuromuscular diseases. Gender, age and site of onset, family history of neuromuscular disorders, disease duration, presence of bulbar and respiratory involvement and presence of extra-motor features were recorded.

### Genetic analysis

The extraction of genomic DNA from peripheral blood samples was performed using established protocols (Qiagen’s FlexiGene DNA Handbook).

Given the length of the study period, genetic analysis was performed with different methods, namely, Sanger sequencing, next-generation sequencing (NGS) MN gene panel, whole-exome sequencing (WES) and short-read whole-genome sequencing (WGS). All patients underwent analysis of the *SOD1* gene, exon 6 of the *TARDBP* gene and exons 13-14-15 of the *FUS* gene, as previously described.^26^ Analysis of *C9orf72* expansion was performed using the Asuragen AmplideX® polymerase chain reaction (PCR)/CE C9orf72 Kit, according to the manufacturer’s instructions.

In a subset of patients, a combination of PCR analysis and subsequent direct Sanger sequencing on an ABI Prism 3130 apparatus was conducted to analyse the coding sequence of *MFN2* (NM_014874.3), as previously described.^18^ The specific primers employed are available upon request. In the other cases, NGS was run on Illumina platforms (MySeq and NextSeq 550), according to the manufacturer’s instructions.

All patients were screened for non-synonymous variants in *MFN2*. All the *MFN2* variants detected were searched for within Franklin using the Genoox website (https://franklin.genoox.com) and screened in the Genome Aggregation Database (gnomAD). The categorization of identified variants into classes (i.e. ‘pathogenic’, ‘likely pathogenic’, ‘VUS’, ‘likely benign’ and ‘benign’) was carried out following the guidelines set forth by the ACMG criteria.^27^

In familial cases, co-segregation analyses were not possible because of the unavailability of genetic material of affected family members, except for Patient 4 (detailed segregation studies are described in Manini *et al*.^26^).

### Statistical analysis

Descriptive statistics was performed where appropriate. Data are expressed in numbers (%) or median (range).

### Protocol approval and informed consent

All patients or their legal representatives signed an informed consent form prior to enrolment. The study was approved by the local Institutional Review Board (Comitato Etico di Milano Area 2 Protocol Number 898_2020bis) and by the ethics committee of the IRCCS Instituto Auxologico Italiano (2023_01_31_01). The study was performed following relevant guidelines and regulations.

## Results

### *MFN2*-amyotrophic lateral sclerosis cohort overview

During the above-mentioned period, 385 amyotrophic lateral sclerosis patients underwent analysis of the *MFN2* gene through either Sanger sequencing (*n* = 10), NGS panel (*n* = 88), WES (*n* = 239) or short-read WGS (*n* = 48). Among them, 23 (5.6%) had a positive family history of amyotrophic lateral sclerosis/frontotemporal dementia.

We identified 19 patients (4.9%) with 12 different non-synonymous variants in *MFN2* (Table 1; Supplementary Table 1) in heterozygous state. None of our patients were homozygous or compound heterozygous for non-synonymous *MFN2* variants. All the variants were absent or rare in public databases (gnomAD allele frequency < 0.01), except for the p.(His20Tyr) variant, which is present in both amyotrophic lateral sclerosis cases and controls in Project MinE data set.^28,29^

**Table 1:** List of rare, non-synonymous MFN2 variants detected in the amyotrophic lateral sclerosis cohort under investigation

#	Chromosomal position	Nucleotide change	Amino acid change	ACMG	gnomAD AF	Previously reported	First description of the patient
1	1:11989226	c.58C>T	p.(His20Tyr)	VUS	7.07e−5	In CMT2A (Bombelli *et al*.^51^; Al-Harbi *et al*.^52^)	Present study
2	1:11992626	c.247T>C	p.(Ser83Pro)	LP	0	Not previously reported	Present study
3	1:11997346	c.524C>T	p.(Ala175Val)	LP	7.95e−6	Not previously reported	Present study
4	1:11997403	c.581A>C	p.(Asp194Ala)	LP	0	Not previously reported	Vinciguerra *et al*.^9^
5	1:12001423	c.839G>A	p.(Arg280His)	P	4.00e−6	In CMT2A (Zuchner *et al*.^11^)	Abati *et al*.^18^
6	1:12001423	c.839G>A	p.(Arg280His)	P	4.00e−6	In CMT2A (Zuchner *et al*.^11^)	Abati *et al*.^18^
7	1:12002028	c.1085C>T	p.(Thr362Met)	P	3.18e−5	In CMT2A - Heterozygous state—mild phenotype (Chung *et al*.^17^) - Compound heterozygous state with either exon 5–6 deletion (Carr *et al*.^53^) or p.(Ala164Val) (Nicholson *et al*.^54^; Calvo *et al*.^55^)—severe and early-onset phenotype	Present study
8	1:12009690	c.2168T>C	p.(Val723Ala)	VUS	0	Not previously reported	Present study
9	1: 12011539	c.2248C>T	p.(His750Tyr)	VUS	0	Not previously reported	Present study

Variants are classified according to the nomenclature Nm_ for the cDNA and Np_ for the protein.

ACMG, American College of Medical Genetics and Genomics; AF, allele frequency; VUS, variants of unknown significance; LP, likely pathogenic; P, pathogenic; CMT2A, Charcot-Marie-Tooth disease type 2A.

Among our cohort, nine patients (2.3%), consisting of five males and four females, were heterozygous for eight different variants which were classified as either pathogenic (*n* = 2), likely pathogenic (*n* = 3) or VUS (*n* = 3) according to the ACMG guidelines and were all missense (Table 1). From this point on, these individuals will be referred to as *MFN2*-amyotrophic lateral sclerosis. Of these, five cases had a positive family history of amyotrophic lateral sclerosis/frontotemporal dementia, and four were classified as sporadic amyotrophic lateral sclerosis. None of the *MFN2*-amyotrophic lateral sclerosis patients tested positive for the pathogenic *C9orf72* gene expansion, nor did they exhibit mutations in the *SOD1* gene or the known mutational hotspots of the *TARDBP* (exon 6) and *FUS* (exons 13-14-15) genes.

**Figure 1:**
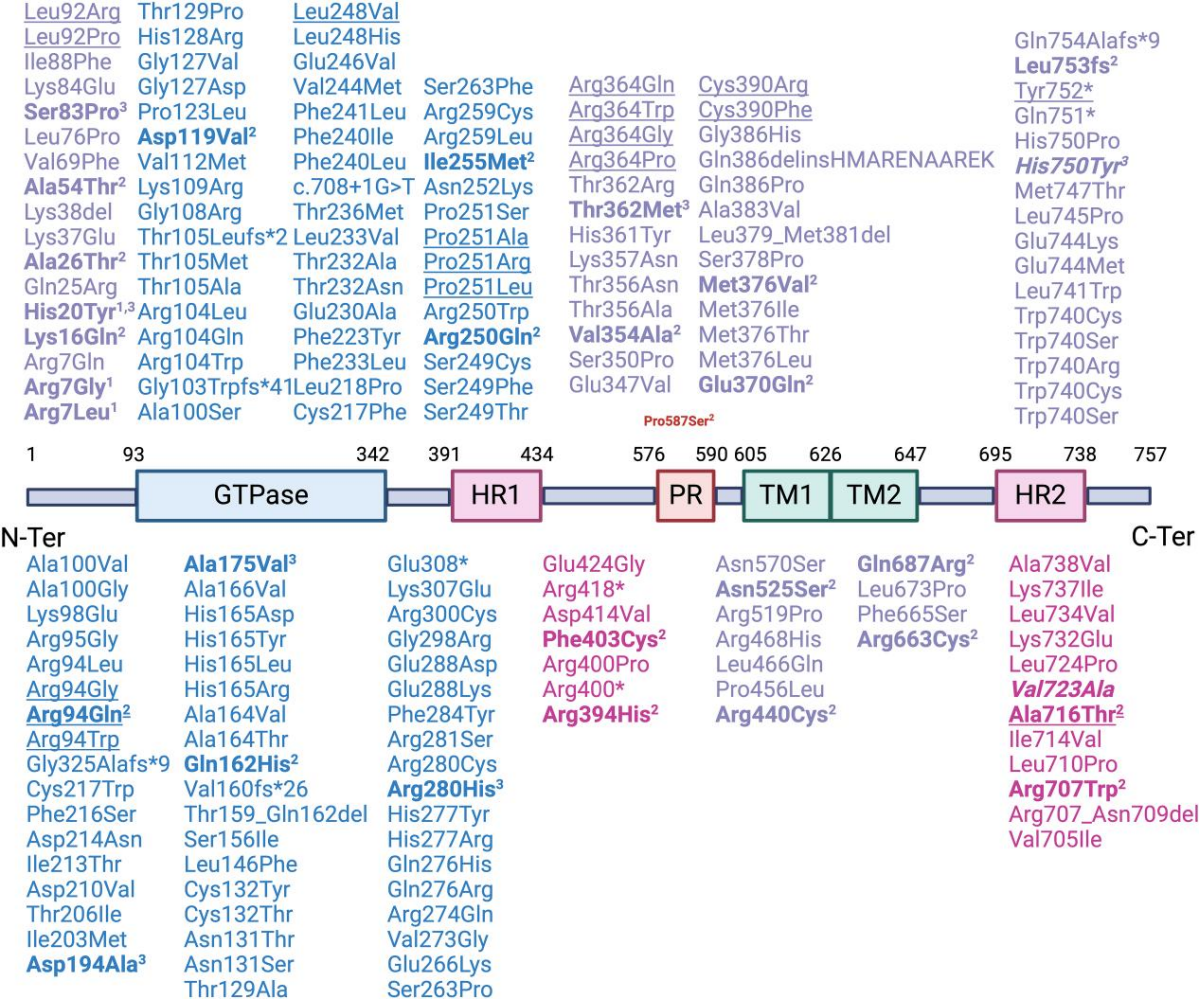
**Schematic representation of the MFN2 protein structure and genetic variants identified so far.**  *MFN2* variants detected in patients and their positions along the mitofusin 2 protein, with data sourced from the Leiden Open Variation Database (LOVD) and https://www.progettomitofusina2.com/portfolio-articoli/database-mutazioni-mfn2/. Superscripts indicate the following: ^1^variants included in the Project MinE database, ^2^variants previously described by Russell *et al*.^10^ and ^3^variants identified in this study. Underlined variants have been detected in Charcot–Marie–Tooth disease type 2A patients with a predominant motor phenotype. Variants in bold have been associated with a MND phenotype. Variants in italics are classified as VUS according to the ACMG.

**Figure 2:**
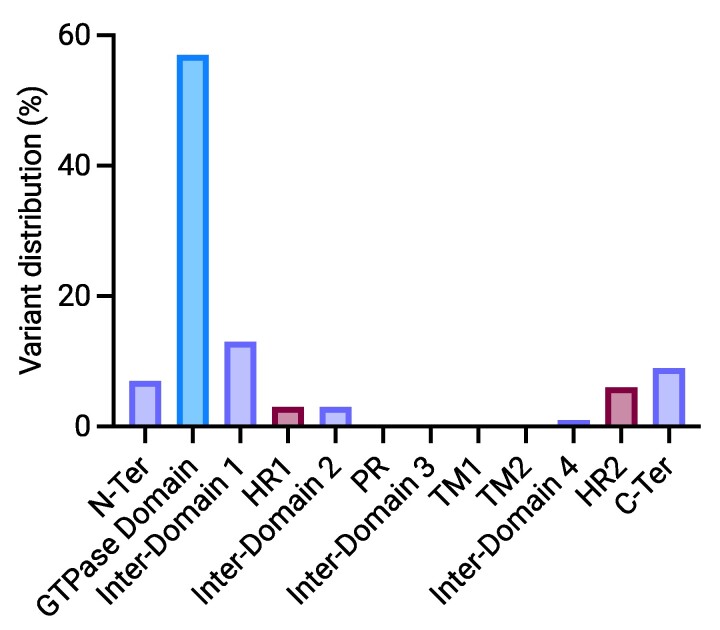
**Distribution of variants across *MFN2* domains.** C-Ter, C-terminal; HR, heptad repeat; N-Ter, N-terminal; PR, proline-rich; TM, transmembrane.

Tables 1 and 2 outline the demographic, genetic and clinical attributes of the *MFN2*-amyotrophic lateral sclerosis patients. Among the identified mutations in the *MFN2* gene, three have already been reported in association with axonal hereditary motor and sensory neuropathy (HMSN), namely, the p.(Arg280His), the p.(His20Tyr) and the p.(Thr362Met). Conversely, the others have never been described in patients. Two unrelated patients carried the p.(Arg280His) mutation. The other variants were retrieved in one patient each. Localization at protein level of variants described so far in *MFN2* is shown in Fig. 1, and distribution across *MFN2* domains is shown in Fig. 2. Variants associated with amyotrophic lateral sclerosis appear to be scattered along the protein sequence rather than showing a specific domain distribution.

**Table 2:** Clinical features of the amyotrophic lateral sclerosis patients harbouring heterozygous, rare, non-synonymous MFN2 variants within the cohort under investigation

#	Amino acid change	Family history	Sex	Age at onset (Ys)	Site of onset	Phenotype	Disease duration (Ys)	UMN signs	LMN signs	Bulbar/respiratory	Non-motor features
1	p.(His20Tyr)	sALS	M	63	UL	Flail arm	>1	+	+	-	-
2	p.(Ser83Pro)	fALS	M	68	UL	Flail arm	3	+	+	+ (NIV 17 months)	-
3	p.(Ala175Val)	sALS	F	29	UL	Classic	12	+	+	+ (PEG 18 months)	-
4	p.(Asp194Ala)	fALS	F	64	LL	ALS-FTD	3	+	+	+	FTD
5	p.(Arg280His)	fALS	M	58	LL	PMA	13	-	+	+ (NIV)	-
6	p.(Arg280His)	fALS	F	69	LL	PMA	8	-	+	-	-
7	p.(Thr362Met)	sALS	M	56	LL	Classic	>3^a^	+	+	-	Sensory symptoms (distal tingling paresthesias) Urinary symptoms (polyuria, incontinence)
8	p.(Val723Ala)	sALS	M	65	LL	Classic	>1^a^	+	+	+ (NIV 10 months)	Sensory symptoms (distal tingling paresthesias)
9	p.(His750Tyr)	fALS	F	51	Bulbar	Bulbar	>1^a^	+	+	-	-

ALS, amyotrophic lateral sclerosis; fALS, familial amyotrophic lateral sclerosis; sALS, sporadic amyotrophic lateral sclerosis; Ys, years; UMN, upper motor neuron; LMN, lower motor neuron; M, male; F, female; NIV, non-invasive ventilation; PEG, percutaneous endoscopic gastrostomy; FTD, frontotemporal dementia; PMA, progressive muscular atrophy; UL, upper limbs; LL, lower limbs.

^a^Follow-up not available.

The median age of onset was 63 years (range 29.1–69.2 years). Most cases (88.8%) had a spinal onset; among them, five patients first experienced symptoms in the lower limbs, while three initially presented with upper limb involvement. In one individual, the disease began with pseudobulbar palsy. We observed significant variability in both the progression and the duration of the disease among the remaining patients (Table 2). Disease duration spanned from less than 1 to 13 years. Notably, two patients have been living with the disease for over a decade (Patients 3 and 5). Patients displayed a huge variety of clinical phenotypes. Two had flail limb syndrome, three had the classical amyotrophic lateral sclerosis phenotype, one had bulbar-onset symptoms and two had progressive muscular atrophy [both patients carrying the p.(Arg280His) mutation]. As the disease progressed, bulbar symptoms emerged in five patients (Patients 2, 3, 4, 5 and 8), while one patient developed frontotemporal dementia (Patient 4). Five patients displayed non-motor symptoms, specifically subjective sensory (Patients 7 and 8) and urinary symptoms (Patient 7), as detailed in Table 2. Six individuals (Patients 1, 3, 4, 5, 6 and 8) exhibited alterations in motor nerve conduction studies, though clinical sensory symptoms and signs were absent and neurophysiological sensory involvement was either absent (Patient 6) or very mild (Patients 4). In five individuals (Patients 1, 4, 5, 7 and 9), motor evoked potentials were available, revealing pathological alterations.

A detailed description of the clinical and neurophysiological characteristics of each patient is provided in the Supplementary material (clinical vignettes; Supplementary Table 2).

## Discussion

Although not consolidated yet, there is a growing body of evidence^8,9^ suggesting an association between variants in the *MFN2* and the amyotrophic lateral sclerosis/frontotemporal dementia spectrum. Moreover, a biological interaction between MFN2 and TDP-43, the main protein involved in amyotrophic lateral sclerosis pathogenesis, has been described in an amyotrophic lateral sclerosis mouse model.^23^ Here, we reported a retrospective study on a large Italian cohort of amyotrophic lateral sclerosis patients aimed at investigating the presence of *MFN2* variants in patients with amyotrophic lateral sclerosis/frontotemporal dementia. Among the 385 amyotrophic lateral sclerosis patients, negative for pathogenic variants in the four most common amyotrophic lateral sclerosis–associated genes, we identified 9 individuals (2.3%) with 8 *MFN2* rare, heterozygous, missense mutations, which were classified as pathogenic, likely pathogenic or VUS according to the ACMG guidelines. Three of them have already been reported in association with axonal HMSN, namely, the p.(His20Tyr), the p.(Arg280His) and the p.(Thr362Met).

The first report of a patient with co-occurrence of Charcot–Marie–Tooth disease type 2A and amyotrophic lateral sclerosis associated with an *MFN2* mutation dates back to 2011.^8^ A second report was published in 2023 by Vinciguerra *et al*.,^9^ detailing one amyotrophic lateral sclerosis/frontotemporal dementia patient with the p.(Asp194Ala) mutation that is now included in our study (Patient 4). In addition, Russell *et al*.^10^ recently performed WGS on 140 amyotrophic lateral sclerosis patients, identifying 21 previously unknown *MFN2* mutations within this cohort. However, to the best of our knowledge, this is the first study to provide a clinical, neurophysiological and molecular characterization of multiple patients affected by amyotrophic lateral sclerosis harbouring *MFN2* variants and the largest work to assess their prevalence.

Within our cohort, age at onset and disease duration were widely variable, in line with literature data,^2^ highlighting the unpredictable nature of amyotrophic lateral sclerosis progression. Indeed, despite median survival in amyotrophic lateral sclerosis spanning between 2 and 4 years, individual courses may show high variability, especially for atypical phenotypes.^2^ Clinical symptom presentation in *MFN2*-amyotrophic lateral sclerosis patients was quite heterogeneous, with the majority exhibiting spinal onset, although upper limb onset and pseudobulbar palsy were also reported. Notably, five patients developed bulbar symptoms during the disease, and one developed frontotemporal dementia. Sensory deficits were observed in five patients, emphasizing the need for a comprehensive clinical assessment of amyotrophic lateral sclerosis patients, including sensory evaluation. Some patients also presented with a concomitant alteration of motor nerve conduction studies. Interestingly, the phenotypic presentation within the family of Patient 4 was diverse, as the proband’s son presented with Charcot–Marie–Tooth disease type 2A with additional mood disturbance. As previously stated, some of the variants reported in this amyotrophic lateral sclerosis cohort have already been described in Charcot–Marie–Tooth disease type 2A patients. Taken all together, these observations might suggest the presence of an *MFN2*-related clinical spectrum, possibly influenced by other genetic modifiers. A growing body of evidence has already demonstrated the presence of an overlap between hereditary neuropathy and MND at a genetic level, with several genes (i.e. *CHCHD10*, *GARS*, *FIG4*, *KIF5A*, *NEFH*, *VCP* and *SPG11*) associated with both amyotrophic lateral sclerosis and Charcot–Marie–Tooth disease type 2.^7^ These considerations notwithstanding, the significance of *MFN2* variants in the context of amyotrophic lateral sclerosis pathogenesis is still unknown and requires further investigations.

Different lines of evidence might suggest the possibility of a link between *MFN2* and amyotrophic lateral sclerosis on both molecular and cellular levels. In their previously mentioned work, Russell *et al*.^10^ employed both *in vitro* and *in vivo* models to understand the specific biological consequences of the novel *MFN2* variants disclosed in their amyotrophic lateral sclerosis cohort. To assess the impact on mitochondrial morphology, the 21 *MFN2* mutations identified were introduced into *Mfn2* KO mouse embryonic fibroblasts (MEFs). Remarkably, none of them could restore normal morphology in *Mfn2* KO MEFs. Some of these mutations also exhibited reduced membrane potential compared to wild-type *MFN2* expression, indicating compromised mitochondrial function. These findings suggest that MFN2-driven mitochondrial dysfunction might potentially lead to MND *in vivo*. Mitochondria are indeed believed to play a significant role in amyotrophic lateral sclerosis development, as evidenced by mitochondrial abnormalities observed both *in vitro* and *in vivo* in multiple amyotrophic lateral sclerosis rodent models.^30^ Swollen mitochondria with unusual *cristae* organization have been detected early in the disease, even before symptom onset in *SOD1* mice.^31^ Inhibiting mitochondrial fission through Drp1 has shown promise in slowing amyotrophic lateral sclerosis progression.^32^ As mentioned above, amyotrophic lateral sclerosis patients with diverse clinical phenotypes, including amyotrophic lateral sclerosis/frontotemporal dementia, pure amyotrophic lateral sclerosis and flail arm syndrome, and patients with axonal Charcot–Marie–Tooth disease have been found to carry missense mutations in the *CHCHD10* gene, encoding the mitochondrial protein CHCHD10.^7,30,33,34^ Interestingly, these mutations have been linked to altered mitochondrial *cristae* structure, impaired stress response and disrupted mitochondrial dynamics, despite no significant changes in canonical fusion and fission protein levels, including MFN2.^35^ The robust body of evidence supporting mitochondrial dysfunction in various neurodegenerative diseases, such as Alzheimer’s and Parkinson’s diseases, underscores the significance of understanding mitochondrial dynamics.^36,37^ The interplay among key regulators of mitochondrial fission and fusion, like mitofusins, adds weight to the role of mitochondrial dynamics in the pathogenesis of these disorders.^36^

Studies using mouse models to explore mutations in mitochondrial fusion and fission regulators, including *Mfn2*, have demonstrated that they lead to mitochondrial depletion in neurites and synapses, resulting in the loss of MN synapses.^38^ Interestingly, amyotrophic lateral sclerosis patients exhibit inclusions containing TDP-43, which are also found in mitochondria within damaged MNs.^38,39^ Russell *et al*.^10^ also explored protein aggregation within the CNS in a *Mfn2* KO zebrafish model, showing that both heterozygous and homozygous *Mfn2* loss-of-function mutations led to increased TDP-43 levels in the hindbrain and cerebellum. Additionally, TDP-43 overexpression was able to rescue the insufficiency of Parkin and its downstream effectors, including MFN2, secondary to progranulin silencing in fibroblasts derived from healthy controls.^40^

TDP-43 has already been shown to negatively affect mitochondrial function and was found in mitochondria.^23,41-44^ Mutant TDP-43 was reported to disrupt mitochondria in mice, leading to impaired transport and membrane potential in neurons.^41,44^ Overexpressing *Mfn2* in these mice restored normal mitochondria and membrane potential. TDP-43 bound mitochondrial mRNAs, especially with amyotrophic lateral sclerosis–causing mutations.^42,43^ Knocking down *TARDBP* reduced MFN2 and blocked its mitochondrial association, improving neuromuscular function in mutant mice.^42,43^ In *Drosophila*, overexpressing Marf was able to mitigate TDP-43-induced disease.^45^

The main genetic cause of amyotrophic lateral sclerosis is a hexanucleotide repeat expansion within *C9orf72*.^46-48^ Recent research demonstrated that the encoded C9orf72 protein plays a role in maintaining the respiratory chain of mitochondria.^49^ C9orf72 was found to reside in the inner mitochondrial membrane, and its loss caused decreased oxidative phosphorylation and ATP production under stress conditions.^50^ Dysfunctional mitochondrial transport and irregular mitochondrial respiration were observed in MNs differentiated from samples of patients with *C9orf72* repeat expansion, potentially involving TDP-43-like effects on mitochondrial mRNAs.^50^

Overall, our findings contribute to a deeper insight into amyotrophic lateral sclerosis genetics and its overlap with other neurological diseases like Charcot–Marie–Tooth disease type 2. We hypothesize that *MFN2*-related clinical syndromes might form a spectrum of MN/axonal degeneration. Functional studies and larger cohorts are needed to further elucidate the role of *MFN2* in MNDs. The creation of big consortia, together with the application of artificial intelligence tools for the analysis of large data sets, may help reach this goal. Furthermore, considering that *MFN2* is a druggable target in neurodegeneration, these results are also interesting from a therapeutic perspective.

In conclusion, the present cohort, together with findings from other works, suggests that *MFN2* variants may play a role in amyotrophic lateral sclerosis pathology, highlighting the relevance of mitochondria and MFN2 in neuronal health.

## Supplementary Material

fcae312_Supplementary_Data

## Data Availability

The data underlying this article will be shared on reasonable request to the corresponding author.
