# Supplementary material for: Investigating the prevalence of MFN2 mutations in amyotrophic lateral sclerosis: insights from an Italian cohort
Source: Brain Commun. 2024 Sep 23;6(5):fcae312. doi: 10.1093/braincomms/fcae312 (PMC11417610; doi:10.1093/braincomms/fcae312)
Supplement: fcae312_Supplementary_Data [file fcae312_supplementary_data.docx]

**Supplementary Material**

**Clinical and neurophysiological vignettes**

Patient 1, a male, initially presented with hand weakness progressing to severe upper limb amyotrophy at the age of 63, with intermittent dysphagia. Neurological assessment at one year revealed tongue fasciculations, marked symmetrical upper limb weakness, and isolated upper motor neuron (UMN) signs, including increased deep tendon reflexes (DTRs) in the lower limbs and bilateral Babinski sign. His retrospectively estimated ALS Functional Rating Scale-Revised (ALSFRS-R) score at 12 months was 32/48. Brain and spinal imaging was unremarkable. Genetic analysis revealed a VUS, p.(His20Tyr), previously reported in Charcot-Marie-Tooth type 2A (CMT2A) patients^1,2^, suggesting a potential genetic role.

At electromyography (EMG), the patient exhibited signs of chronic and active denervation in all body regions, particularly prominent in the cervical and thoracic segments. Nerve conduction studies (NCS) showed reduced compound motor action potential (CMAP) amplitudes and motor conduction velocity (MCV) in the upper limbs. No abnormality in the sensory parameters was detected. Motor evoked potentials (MEPs) recorded from the abductor hallucis showed marked decrease in amplitude and increased central motor conduction latency in the lower limbs. Somatosensory-evoked potentials (SSEPs) were normal for the upper limbs (stimulation of median nerves) but not recorded for the lower limbs.

Patient 2, a male, developed progressive and asymmetric upper limb weakness and fasciculations at the age of 68. Examination revealed signs of involvement of both upper and lower MNs, including in the bulbar segment. Spinal Magnetic Resonance Imaging (MRI) showed cervical spondylosis, while brain MRI was not performed at the patient’s request. Respiratory problems requiring non-invasive ventilation (NIV) occurred around 17 months after onset. The ALSFRS-R score at two years was 34 out of 48. Genetic testing identified the likely pathogenic p.(Ser83Pro) mutation in the *MFN2* gene, previously unreported in the literature and classified as likely pathogenic.

Patient 3, a 28-year-old female, initially presented with unilateral hand weakness and subsequent unilateral lower limb rigidity. She had no family history of motor neuron or other neuromuscular diseases. Brain MRI revealed corticospinal tract hyperintensity, while spinal imaging and routine cerebrospinal fluid (CSF) analysis were unremarkable. Disease duration exceeded 10 years and her clinical course was characterized by the occurrence, approximately four years after disease onset, of signs of bulbar involvement and loss of independent walking. After more than seven years, her ALSFRS-R score was 20 out of 48, accompanied by significant UMN and LMN signs (severe weakness and amyotrophy together with spasticity and polykinetic DTRs) in the bulbar, cervical and lumbar regions, and isolated involvement of LMN in the thoracic region. Genetic analysis identified a likely pathogenic p.(Ala175Val) variant in the *MFN2* gene, previously unreported in the literature and classified as likely pathogenic. She required a percutaneous endoscopic gastrostomy (PEG) around ten years after disease onset and died 18 months later.

Motor NCSs performed in this patient showed a reduced CMAP amplitude in the peroneal nerves and absent right ulnar CMAP. EMG displayed chronic neurogenic changes in both the upper and lower limb muscles, with active denervation in the right medial gastrocnemius.

Patient 4, a 64-year-old female, displayed a complex phenotype with both spinal involvement and frontotemporal dementia (FTD). The identified p.(Asp194Ala) variant is classified as likely pathogenic and was not reported previously until noted by us.^3^ She experienced disease onset in the lower limbs, which was further complicated by bulbar involvement and behavioral-affective disturbances consistent with FTD within three years. This case was particularly notable for its neuropsychiatric component and familial trend, as her 41-year-old son and other relatives presented with similar symptoms, namely psychiatric disorders and gait disturbances in one sister and a maternal cousin. Neurological examination showed pseudobulbar and pyramidal features in conjunction with LMN features such as tongue fasciculations and severe diffuse muscle wasting. Limb weakness was more pronounced distally with complete loss of ankle dorsiflexion and plantar flexion. DTRs were brisk in the upper limbs and absent in the lower limbs, with bilateral Hoffmann and Babinski signs. Gait was markedly paraparetic. Mini Mental State Examination (MMSE) showed a score of 22/30 with a dysexecutive syndrome. ALSFRS-R score was 35/48.

NCSs revealed a significant reduction of CMAP amplitudes in the upper and lower limbs, while needle EMG examination revealed widespread active positive sharp waves and fasciculation potentials in all bulbar, cervical, thoracic and lumbar segments, associated with chronic denervation. MEPs from posterior tibial nerves were absent.

Patient 5, a 58-year-old male, exhibited initial symptoms in the lower limbs. The patient's sister had a similar phenotype, suggesting a genetic component to the disease presentation. As documented by Abati *et al.* in 2022^4^, symptoms began with lower limb weakness which progressively advanced over 13 years. He then developed nocturnal hypoventilation, for which he was prescribed NIV through a continuous positive airway pressure (CPAP) device. At the time of the last review, neurological examination disclosed LMN signs, including severe weakness and atrophy of the lower limbs, and a high-frequency upper limb tremor. The diagnosis was further substantiated by the likely pathogenic p.(Arg280His) variant associated with CMT2A.^5^ Despite the duration of the disease, no additional extra-motor features were reported in this case. At the time of review, ALSFRS-R score was 42/48.

EMG/ NCSs showed diffuse signs of chronic denervation in the four limbs, including proximal muscles, paraspinal and abdominal muscles, and signs of active denervation and pseudomyotonic discharges in both the cervical and lumbar segments. Additionally, he presented reduced CMAP and slightly decreased MCV in the posterior tibial nerves.

Patient 6 was a 76-year-old female at the time of last review. She had a positive family history, with her brother reporting similar symptoms, but he never got tested and is now deceased. She tested positive for the p.(Arg280His) variant, consistent with familial CMT2A. Her symptoms began at the age of 48 with weakness and atrophy in the lower limbs, which then progressed to the upper limbs and the bulbar region. Neurological examination disclosed dysphagia, moderate distal upper limb weakness and severe weakness and atrophy in the lower limbs. No extra-motor features were observed during the course of the disease. Her ALSFRS-R score was 40/48.

EMG/NCSs demonstrated the presence of signs of active and chronic denervation at both proximal and distal muscles in the lower limbs. She also presented severely reduced CMAPs in the lower limbs.

Patient 7 initially presented with progressive asymmetrical lower limb weakness at age 56. Three years later, a neurological examination revealed a complex picture with involvement of both UMN and LMN in the four limbs, including mild muscle weakness and atrophy, increased DTRs, spastic gait, and distal tingling paresthesias. Urinary problems, including polyuria and occasional incontinence, were reported. Routine CSF analysis and central nervous system (CNS) imaging showed no significant abnormalities, aside from mild cerebellar and thoracic spinal cord atrophy and mild spondylosis. A retrospective ALSFRS-R score of 43/48 was estimated at three years from symptom onset. Genetic testing in this sporadic case identified a p.(Thr362Met) mutation, classified as pathogenic, and already described in association with both mild, late-onset^6^ and severe early-onset CMT2A, the latter when in compound heterozygosity.^7–9^

In this patient, EMG findings indicated chronic neurogenic changes in bulbar, cervical, thoracic and lumbar segments, while active denervation was only present in cervical and lumbar segments. Sensory and motor conduction parameters were normal. Central MEPs were absent in the lower limbs, while a mild decrease in amplitude was observed in the right upper limb. SSEPs revealed an absence of spinal and cortical potentials for the lower limbs, being unremarkable in the upper limbs.

Patient 8, a 65-year-old male, initially complained of proximal right lower limb weakness, leading to frequent falls. Within a month, symptoms progressed to involve the ipsilateral upper limb and distal tingling paresthesias appeared in the affected lower limb. A neurological examination at 7 months revealed a complex picture with signs of involvement of both UMN and LMN in the four limbs, including spasticity, increased DTRs, fasciculations and muscle wasting, along with abdominal muscle weakness. Notably, the disease was more severe on the side of onset, with lower limbs showing more prominent UMN signs and upper limbs displaying predominantly LMN signs. Medical history was positive for neoplasia (head and trunk basal cell carcinomas and colorectal dysplasia), urinary urgency, and prostatic adenoma. Brain MRI and routine CSF examination were unremarkable, muscle biopsy showed neurogenic involvement, and the ALSFRS-R score decreased rapidly from 28 to 18 within 4 months. NIV was required 10 months after onset, but the patient was lost to follow-up at 14 months. Genetic testing identified a novel p.(Val723Ala) variant classified as a VUS.

EMG findings showed chronic neurogenic denervation and reduced MUP recruitment in the bulbar, cervical and lumbar regions, with active denervation recorded in the upper limbs. NCSs displayed a markedly reduced amplitude of the CMAPs in the upper limbs, with normal sensory conductions.

Patient 9, a 51-year-old female, initially presented with pseudobulbar palsy and progressive dysphagia for liquids. Within a year, she developed dysarthria and limb weakness in the four limbs. A neurological exam at 15 months revealed both UMN and LMN involvement. Bulbar signs included atrophic tongue with fasciculations and jaw clonus, while upper limbs showed mild distal amyotrophy, increased DTRs, and spasticity. Lower limbs had isolated UMN involvement. Brain MRI showed non-specific white matter changes, while spinal cord MRI was not performed. Routine CSF analysis was unremarkable. The patient had a positive family history for neuromuscular disorders. Her father developed a progressive lower limb weakness at 48 years of age, followed by upper limb involvement; he never received genetic testing and is now deceased. He died during sleep at age 62, after being diagnosed with a laryngeal tumor two years earlier. Patient’s maternal cousin was reported to have a progressive paresis of lower and upper limbs. None of the affected relatives underwent genetic analysis. Genetic testing in the proband identified the novel p.(His750Tyr) variant, classified as a VUS. A different amino acid substitution at the same codon (p.(His750Pro)) was previously reported as pathogenic in CMT2A cases.^10^ At the last visit, 16 months after onset, respiratory function and swallowing were not severely compromised, with no need for NIV or PEG.

EMG revealed chronic neurogenic involvement in the right upper limbs and the bulbar region. MEPs indicated corticospinal involvement in the right limbs, while SSEPs were not available for assessment.

**Supplementary table 1. Benign and likely benign variants in the *MFN2* gene identified in our cohort.** Other rare coding variants in *MFN2* not fulfilling the American College of Medical Genetics and Genomics (ACMG) criteria for “pathogenic”, “likely pathogenic”, or “variant of unknown significance” (VUS). All the variants are absent in Project Mine.

| **Genomic position** | **Nucleotide change** | **Amino acid change** | **Number of patients** | **ACMG criteria** | **Previously reported** | **GnomAD AF** |
| --- | --- | --- | --- | --- | --- | --- |
| 1:12001425 | c.841T>A | p.(Cys281Ser) | 1 | Likely benign | Brožková et al, 2013^11^: three families with axonal HMSN (proband and healthy relatives) | 0 |
| 1:12001426 | c.842G>C | p.(Cys281Ser) | 1 | Likely benign | Brožková et al, 2013^11^: three families with axonal HMSN (proband and healthy relatives) | 0.0001379 |
| 1:12001476 | c.892G>A | p.(Gly298Arg) | 3 | Benign | Lawson et al., 2005^12^: nucleotide substitution c.891G>A, resulting in the same aminoacidic change, was found in 3% of controls | 0.002162 - 1 homozygous subject in GnomAD |
| 1:12004835 | c.1403G>A | p.(Arg468His) | 3 | Benign | Engelfried et al., 2006^13^: detected in 1/260 control chromosomes | 0.002545 - 3 homozygous subjects in GnomAD |
| 1:12009635 | c.2113G>A | p.(Val705Ile) | 2 | Benign | Albulym et al., 2013^14^: present in 1 affected family member only and absent in the other affected members; found in 5/57 healthy controls. | 0.005862 - 14 homozygous subjects in GnomAD |

**Supplementary** **Table 2. Neurophysiological data of patients carrying *MFN2* variants**

| **Case ID** | **EMG** | | | | **ENG - Motor** | | | | **ENG - Sensory** | | | | **MEPs** | | | | **SSEPs** | | | |
| --- | --- | --- | --- | --- | --- | --- | --- | --- | --- | --- | --- | --- | --- | --- | --- | --- | --- | --- | --- | --- |
|  | **Bulbar** | **Cervical** | **Trunk** | **Lumbar** | **Upper limbs** | | **Lower limbs** | | **Upper limbs** | | **Lower limbs** | | **Upper limbs** | | **Lower limbs** | | **Upper limbs** | | **Lower limbs** | |
|  |  |  |  |  | **R** | **L** | **R** | **L** | **R** | **L** | **R** | **L** | **R** | **L** | **R** | **L** | **R** | **L** | **R** | **L** |
| 1 | P | P | P | P | P | P | N | N | N | N | N | N | U | U | P | P | N | N | / | / |
| 2 | / | / | / | / | / | / | / | / | / | / | / | / | / | / | / | / | / | / | / | / |
| 3 | / | P | / | P | P | / | P | P | N | / | N | / | / | / | / | / | / | / | / | / |
| 4 | P | P | P | P | P | P | P | P | P | P | P | P | P | P | P | P | / | / | / | / |
| 5 | N | P | P* | P | N | N | P | P | N | N | N | N | P | P | P | P | / | / | / | / |
| 6 | N | N | N | P | N | N | P | P | N | N | N | N | / | / | / | / | / | / | / | / |
| 7 | P* | P | P* | P | N | N | N | N | N | N | N | N | U | N | P | P | N | N | P | P |
| 8 | P* | P | / | P* | -- | P | P | P | N | N | N | N | / | / | / | / | / | / | / | / |
| 9 | P* | P* | / | N | N | N | N | N | N | N | N | N | P | N | P | N | / | / | / | / |
| **Abbreviations**: EMG electromyography, MEPs motor evoked potentials, SSEPs somatosensory evoked potentials, R right, L left, M motor, S sensory, P pathologic, N normal or w/i normal limits, U undefined (data of uncertain significance), / not performed/not available.  The exams included in the table were the most representative for the patient, therefore they may not correspond to the same disease timing.  *Reduced recruitment only (no active denervation) | | | | | | | | | | | | | | | | | | | | |

**References**

1. Al-Harbi TM, Abdulmana SO, Bashir S, Dridi W. Novel MFN2 Missense Mutation Induces Hereditary Axonal Motor and Sensory Neuropathy in a Saudi Arabian Family. *J Clin Neuromuscul Dis*. 2019;21(1):25-29. doi:10.1097/CND.0000000000000244

2. Bombelli F, Stojkovic T, Dubourg O, et al. Charcot-Marie-Tooth disease type 2A: from typical to rare phenotypic and genotypic features. *JAMA Neurol*. 2014;71(8):1036-1042. doi:10.1001/jamaneurol.2014.629

3. Vinciguerra C, Di Fonzo A, Monfrini E, et al. Case report: Asp194Ala variant in MFN2 is associated with ALS-FTD in an Italian family. *Front Genet*. 2023;14. doi:10.3389/FGENE.2023.1235887

4. Abati E, Manini A, Velardo D, et al. Clinical and genetic features of a cohort of patients with MFN2-related neuropathy. *Sci Rep*. 2022;12(1):6181. doi:10.1038/S41598-022-10220-0

5. Züchner S, De Jonghe P, Jordanova A, et al. Axonal neuropathy with optic atrophy is caused by mutations in mitofusin 2. *Ann Neurol*. 2006;59(2):276-281. doi:10.1002/ana.20797

6. Chung KW, Kim SB, Park KD, et al. Early onset severe and late-onset mild Charcot-Marie-Tooth disease with mitofusin 2 (MFN2) mutations. *Brain*. 2006;129(Pt 8):2103-2118. doi:10.1093/brain/awl174

7. Carr AS, Polke JM, Wilson J, et al. MFN2 deletion of exons 7 and 8: founder mutation in the UK population. *J Peripher Nerv Syst*. 2015;20(2):67-71. doi:10.1111/JNS.12117

8. Calvo J, Funalot B, Ouvrier RA, et al. Genotype-phenotype correlations in Charcot-Marie-Tooth disease type 2 caused by mitofusin 2 mutations. *Arch Neurol*. 2009;66(12):1511-1516. doi:10.1001/ARCHNEUROL.2009.284

9. Nicholson GA, Zhu D, Magdelaine C, et al. Severe early-onset axonal neuropathy with homozygous and compound heterozygous MFN2 mutations. *Neurology*. 2008;70(19):1678-1681. doi:10.1212/01.WNL.0000311275.89032.22

10. Feely SME, Laura M, Siskind CE, et al. MFN2 mutations cause severe phenotypes in most patients with CMT2A. *Neurology*. 2011;76(20):1690-1696. doi:10.1212/WNL.0b013e31821a441e

11. Brožková DŠ, Posádka J, Laššuthová P, et al. Spectrum and frequencies of mutations in the MFN2 gene and its phenotypical expression in Czech hereditary motor and sensory neuropathy type II patients. *Mol Med Rep*. 2013;8(6):1779-1784. doi:10.3892/MMR.2013.1730

12. Lawson VH, Graham B V., Flanigan KM. Clinical and electrophysiologic features of CMT2A with mutations in the mitofusin 2 gene. *Neurology*. 2005;65(2):197-204. doi:10.1212/01.WNL.0000168898.76071.70

13. Engelfried K, Vorgerd M, Hagedorn M, et al. Charcot-Marie-Tooth neuropathy type 2A: novel mutations in the mitofusin 2 gene (MFN2). *BMC Med Genet*. 2006;7. doi:10.1186/1471-2350-7-53

14. Albulym OM, Zhu D, Reddel S, Kennerson M, Nicholson G. The MFN2 V705I Variant Is Not a Disease-Causing Mutation: A Segregation Analysis in a CMT2 Family. *J Neurodegener Dis*. 2013;2013:1-5. doi:10.1155/2013/495873
